# Drivers of Young People’s Self-Harm Presentations to Emergency Departments: A Berkshire Service Evaluation

**DOI:** 10.1192/j.eurpsy.2026.10564

**Published:** 2026-07-31

**Authors:** T. Mehdi, M. Duffield, E. Hall

**Affiliations:** Child and Adolescent Mental Health, Berkshire Healthcare Foundation NHS Trust, Reading, United Kingdom

## Abstract

**Introduction:**

Epidemiological data indicate that **lifetime** self-harm is reported by approximately **16%** of adolescents (Farkas *et al*.ECAP 2023 (10):3439-3458). A proportion present to hospital emergency departments (A&E) in a crisis self-harm presentation such as overdose, attempted hanging, self-injury or jumping. These attendances are often misinterpreted as evidence of a primary mental illness; in many cases, factors other than mental illness are the drivers of crisis. Over-attributing crisis to “mental illness” can prompt reactive interventions that miss root causes, waste resources, and fail to improve emotional stability or functioning in children and young people (CYP).

**Objectives:**

Evaluate crisis presentations to Berkshire Child and Adolescent Mental Health (CAMHS) Rapid Response Team (1 Feb–30 Apr 2022); describe demographics and presenting features; identify drivers of crisis; and outline implications for multi-agency responses and care to support optimal outcomes for CYP.

**Methods:**

Mixed-methods retrospective case-note review of all CYP referred to Berkshire CAMHS Rapid Response from two acute hospitals after A&E mental-health presentations (Feb–Apr 2022). Data source: electronic records. Qualitative: narratives describing the main contributing factor for attendance were coded and thematically analysed to identify patterns. Quantitative data included age, sex, presenting reason, self-harm type, autism/ADHD diagnosis or waitlist, other psychiatric diagnoses, CAMHS involvement, other service input, family adversity and education. Inter-rater reliability was checked on 10 cases, with unclear cases reviewed by two clinicians to reach consensus.

**Results:**

213 self-harm crisis presentations were evaluated. Crises were largely associated with environmental factors, led by school (37.5%) and family factors (23.5%). Overall, 71% did not require ongoing CAMHS input (29% did). 55% were referred to other services, 39% were already receiving support, and 47% required multi-agency liaison. Neurodevelopmental needs were common: 22% had a diagnosis of autism or ADHD and a further 28% were awaiting assessment/referred on—indicating potentially 50% of CYP with neurodevelopmental profiles.

**Image 1:**
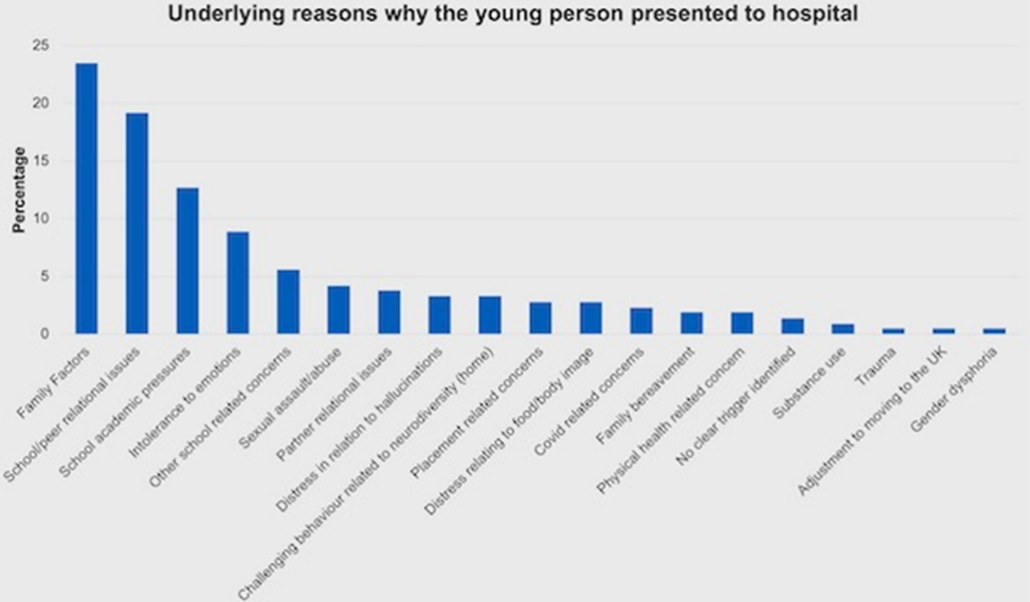
Image 1: Long description.

**Image 2:**
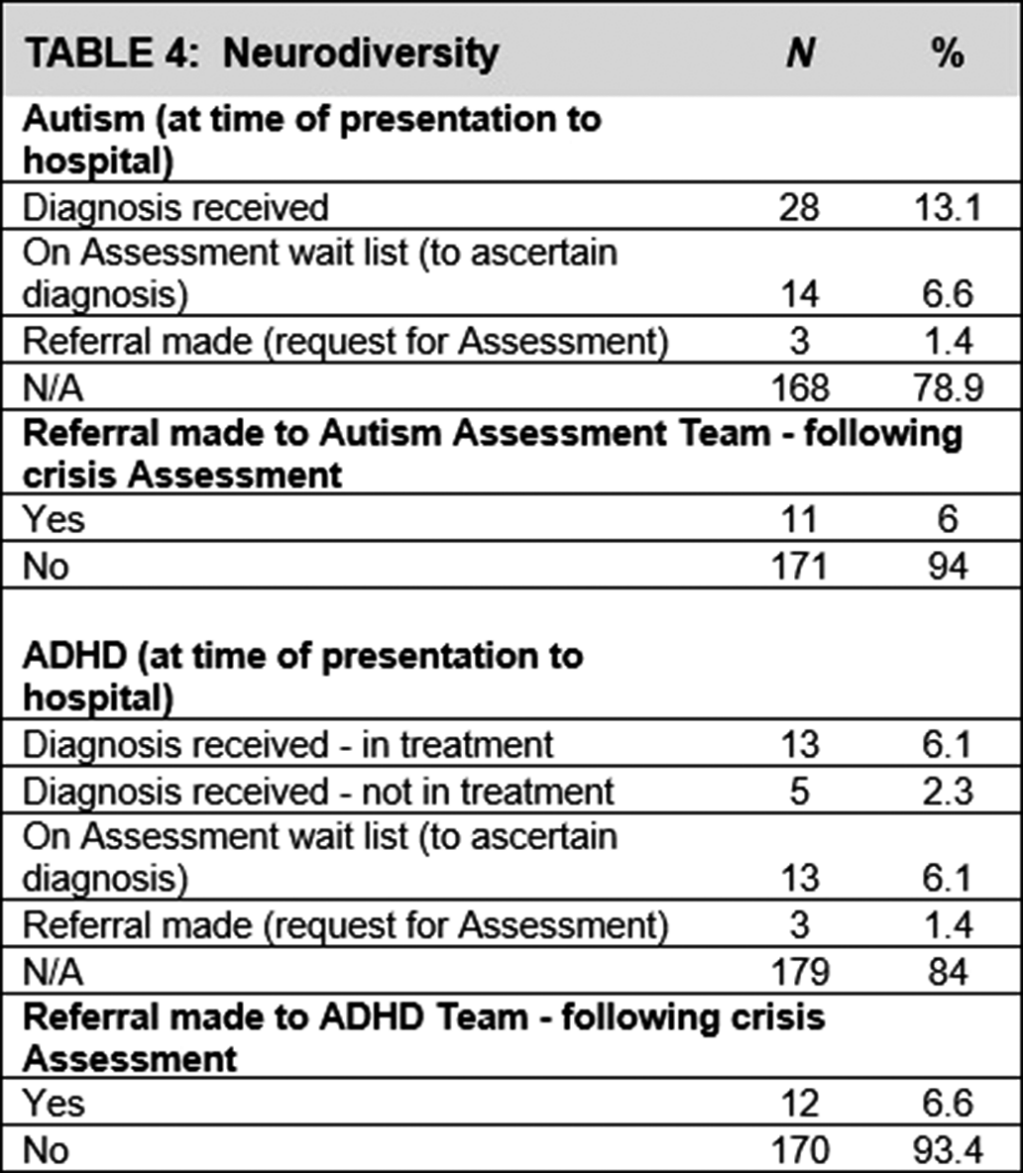
Image 2: Long description.

**Image 3:**
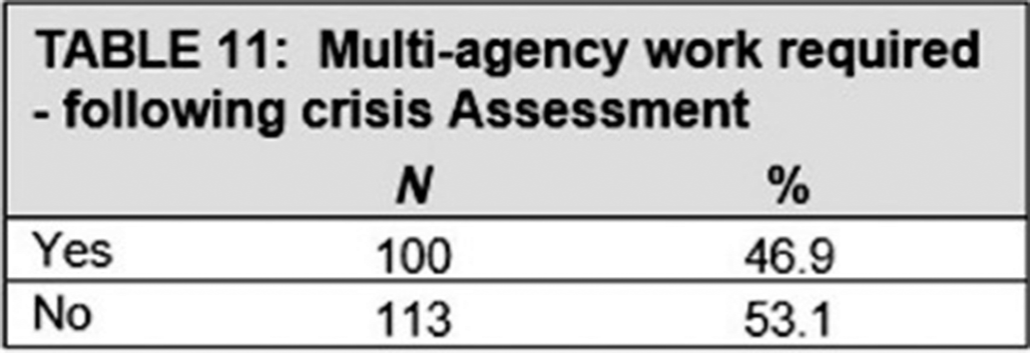

**Conclusions:**

Emergency and crisis services should recognise that self-harm presentations in children and young people are heterogeneous: some presentations reflect mental illness, but many are driven by environmental stressors or unmet neurodevelopmental needs. Risk-led and premature mental-health interventions risk missing root causes, wasting resources and yielding poor outcomes. Responses from acute services should prioritise a holistic, needs-led assessment to distinguish psychiatric illness from contextual/developmental drivers. Where these predominate, CAMHS should provide targeted liaison to partner agencies to activate and steer relevant interventions addressing social, family, educational and developmental needs.

**Disclosure of Interest:**

None Declared

## Long descriptions

### Image 1: Long description

The vertical Y axis is labeled Percentage and ranges from 0 to 25 in increments of 5. The horizontal X axis lists twenty categories. The data shows a sharp decline from the first few categories followed by a long tail of lower-frequency reasons.

* Family Factors: approximately 23.5 percent

* School or peer relational issues: approximately 19 percent

* School academic pressures: approximately 12.5 percent

* Intolerance to emotions: approximately 9 percent

* Other school related concerns: approximately 5.5 percent

* Sexual assault or abuse: approximately 4 percent

* Partner relational issues: approximately 3.8 percent

* Distress in relation to hallucinations: approximately 3.2 percent

* Challenging behaviour related to neurodiversity home: approximately 3.2 percent

* Placement related concerns: approximately 2.8 percent

* Distress relating to food or body image: approximately 2.8 percent

* Covid related concerns: approximately 2.2 percent

* Family bereavement: approximately 1.8 percent

* Physical health related concern: approximately 1.8 percent

* No clear trigger identified: approximately 1.2 percent

* Substance use: approximately 0.8 percent

* Trauma: approximately 0.5 percent

* Adjustment to moving to the U K: approximately 0.5 percent

* Gender dysphoria: approximately 0.5 percent

Navigate back to Image 1:.

### Image 2: Long description

The table contains three columns: Category, N, and percentage. It is divided into two primary sections.

Section 1: Autism at time of presentation to hospital.

* Diagnosis received: 28, 13.1 percent.

* On Assessment wait list to ascertain diagnosis: 14, 6.6 percent.

* Referral made request for Assessment: 3, 1.4 percent.

* N / A: 168, 78.9 percent.

* Referral made to Autism Assessment Team following crisis Assessment: Yes is 11, 6 percent; No is 171, 94 percent.

Section 2: A D H D at time of presentation to hospital.

* Diagnosis received in treatment: 13, 6.1 percent.

* Diagnosis received not in treatment: 5, 2.3 percent.

* On Assessment wait list to ascertain diagnosis: 13, 6.1 percent.

* Referral made request for Assessment: 3, 1.4 percent.

* N / A: 179, 84 percent.

* Referral made to A D H D Team following crisis Assessment: Yes is 12, 6.6 percent; No is 170, 93.4 percent.

Navigate back to Image 2:.

